# Habitual sodium intake and metabolic abnormalities in Korean adults with obesity: a cross-sectional analysis of a prospective cohort

**DOI:** 10.1186/s12889-026-28903-y

**Published:** 2026-08-06

**Authors:** Soo-Hyun Park, Minyoung Lee, Hee-Joo Kang, Jin-Hee Kim, Jin-Taek Hwang, Jina Chung, Hyojee Joung, Sungha Park, Jae-Ho Park

**Affiliations:** 1 Division of Food Functionality Research, Korea Food Research Institute, Wanju, Republic of Korea; 2 Department of Internal Medicine, Division of Endocrinology and Metabolism, Severance Hospital, Yonsei University College of Medicine, Seoul, Republic of Korea; 3 Institute of Endocrine Research, Yonsei University College of Medicine, Seoul, Republic of Korea; 4 Department of Biomedical Science, Seoul National University Graduate School, Seoul, Republic of Korea; 5 Department of Public Health, Graduate School of Public Health, Seoul National University, Seoul, Republic of Korea; 6 Institute of Health and Environment, Seoul National University, Seoul, Republic of Korea; 7 Department of Internal Medicine, Division of Cardiology, Severance Hospital, Yonsei University College of Medicine, Seoul, Republic of Korea; 8 Integrative Research Center for Cerebrovascular and Cardiovascular Diseases, Yonsei University College of Medicine, Seoul, Republic of Korea

**Keywords:** Sodium, Obesity, Metabolic disease, Lipid, Cohort

## Abstract

**Background:**

Sodium intake is well recognized for its contribution to hypertension, but growing evidence suggests broader metabolic effects, particularly among individuals with obesity who are vulnerable to metabolic disturbances. This study investigated the cross-sectional associations between habitual sodium intake and metabolic diseases and related markers using baseline data from a prospective cohort of Korean adults with obesity.

**Methods:**

Participants with obesity (BMI ≥ 25 kg/m^2^) and at least one metabolic condition were recruited from Severance Hospital, Seoul, Republic of Korea. Sodium intake was assessed using a food frequency questionnaire, and associations with diabetes, dyslipidemia, hypertension, and cardiovascular disease were evaluated using multivariable regression models. Mean differences in metabolic markers across sodium tertiles were examined using analysis of covariance with sex stratification.

**Results:**

Of the 633 individuals enrolled, 487 with obesity were included in the final analysis after excluding non-obese participants and those with implausible energy intake. No statistically significant associations were observed between sodium intake and the prevalence of type 2 diabetes, dyslipidemia, hypertension, or cardiovascular disease. In sex-stratified biomarker analyses, LDL-C, non-HDL-C, CRI-I, and CRI-II differed across sodium tertiles among women, whereas no such differences were observed in men.

**Conclusions:**

In Korean adults with obesity and pre-existing metabolic conditions, increasing sodium intake was not significantly associated with higher prevalence of major metabolic diseases. In exploratory sex-stratified analyses, several atherogenic lipid markers differed across sodium intake tertiles among women only. This within-women pattern should be regarded as hypothesis-generating and requires confirmation in future longitudinal analyses of this cohort.

**Supplementary Information:**

The online version contains supplementary material available at https://doi.org/10.1186/s12889-026-28903-y.

## Introduction

Chronic metabolic diseases, such as type 2 diabetes mellitus (T2DM), dyslipidemia, and hypertension, are multifactorial conditions influenced by the complex interplay of behavioral, physiological, and dietary factors. If inadequately managed, these conditions can lead to more advanced outcomes such as cardiovascular disease (CVD), reflecting a later stage of metabolic dysfunction and one of the most critical contributors to adverse health outcomes globally [1–3]. The risk of chronic metabolic diseases is substantially heightened in individuals with obesity, and sustained unhealthy lifestyle behaviors, including unbalanced nutrition, insufficient physical activity, and dietary imbalances, can accelerate the progression of metabolic dysfunction [4–6]. Therefore, understanding the pathophysiological characteristics and habitual dietary patterns of individuals with obesity is essential for identifying modifiable risk factors and preventing chronic metabolic diseases.

Among various nutritional factors, sodium has been most actively investigated in relation to metabolic diseases [7–11]. Sodium is widely recognized for its association with elevated blood pressure, and several studies established the causal role of sodium in the development of hypertension [12, 13]. International guidelines, such as those from the World Health Organization (WHO), have formalized sodium intake recommendations based on this evidence [14]. Beyond its hypertensive effects, sodium can influence a broader range of metabolic outcomes, including lipid metabolism, insulin resistance, adiposity, hepatic steatosis, and systemic inflammation [7–9]. These effects are believed to involve various mechanisms, such as alterations in hormonal regulation, fluid balance, and adipocyte function [10, 11]. Despite this biological plausibility, the relationship between sodium intake and metabolic outcomes remains insufficiently studied, particularly in human populations. As sodium intake frequently exceeds the recommended thresholds among high-risk groups, especially those with obesity [15, 16], evaluating population-based differences in intake levels could represent a more informative approach to understanding metabolic risk.

To address this gap, we examined the relationship between habitual sodium intake and metabolic health in a Korean adult cohort with a high proportion of individuals at elevated metabolic risk, including those with obesity. Specifically, we assessed the associations of sodium intake with the prevalence of chronic metabolic diseases and related clinical, biochemical, anthropometric, and imaging-based parameters.

## Materials and methods

### Study design and participants

This study used baseline data from the Nutriomics and Artificial Intelligence Nutrition Obesity Cohort (NAINOC), a prospective cohort with five scheduled follow-up assessments designed to examine associations between dietary patterns and chronic metabolic diseases in Korean adults with obesity [17]. This study is reported in accordance with the Strengthening the Reporting of Observational Studies in Epidemiology (STROBE) guidelines [18]. Using these data, we investigated the cross-sectional associations between habitual sodium intake and the prevalence of metabolic diseases and related cardiometabolic markers at cohort entry. Individuals visiting Yonsei University College of Medicine, Severance Hospital (Seoul, South Korea) were targeted for enrollment. Sample size calculation was previously described in the study protocol [17].

**Fig. 1:**
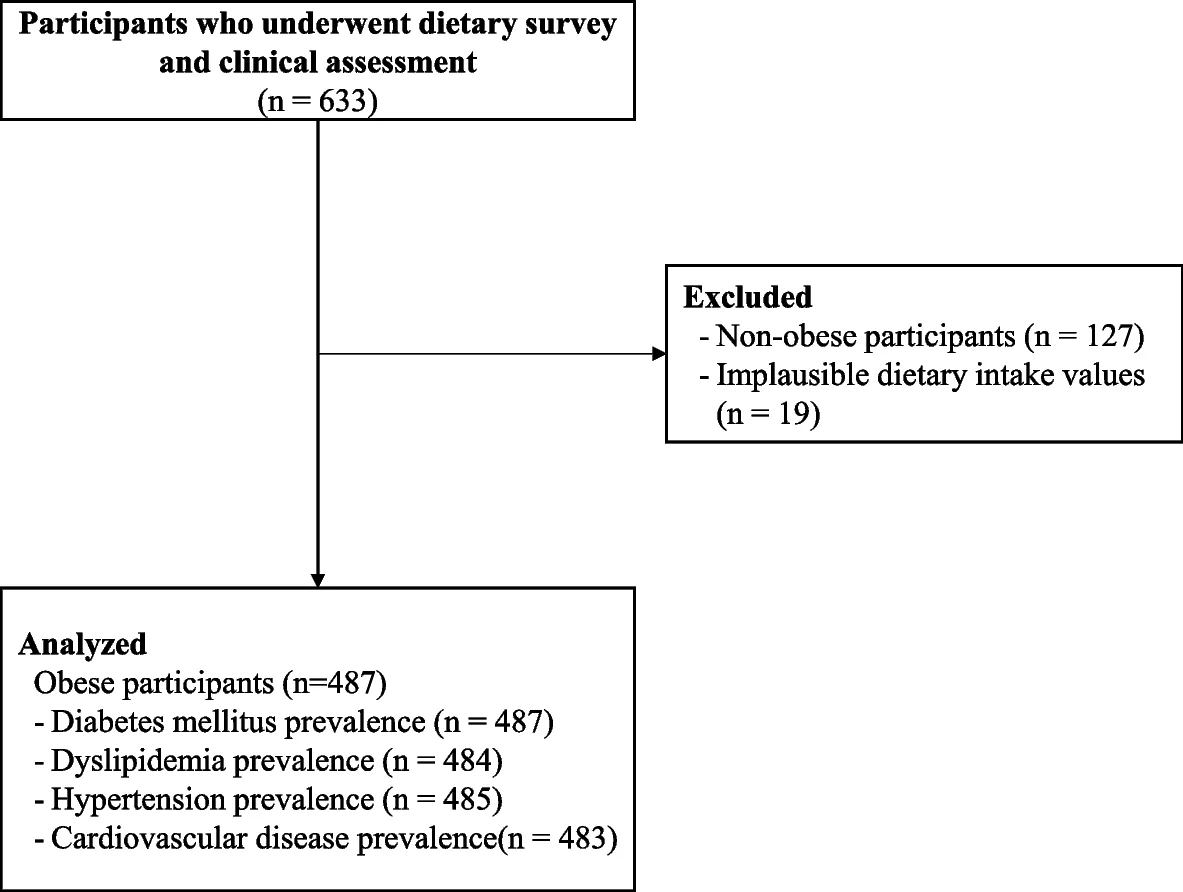
Participant flow diagram

The cohort enrolled two groups: an obese group with at least one chronic metabolic disease and a non-obese control group, as described in the cohort protocol. The present analyses were restricted to the obese group, as the primary aim of this study was to examine sodium-metabolic health associations in individuals at high metabolic risk. Eligible participants in the obese group were aged ≥ 19 years with obesity [body mass index (BMI) ≥ 25 kg/m^2^] and at least one chronic metabolic disease [e.g., hypertension, T2DM, CVD, metabolic syndrome]. Participants meeting any of the following criteria were excluded: (1) life expectancy of fewer than 6 months because of underlying diseases excluding CVD; (2) pregnancy; (3) organ transplantation within the past 3 months; (4) current treatment for acute rejection of a transplanted organ; (5) treatment for acute coronary syndrome within the past 6 months; (6) treatment for acute cerebrovascular disease within the past 6 months; (7) diagnosis of type 1 diabetes mellitus; (8) use of steroids or hormone replacement therapy. The participant flow is summarized in Fig. 1.

### Ethical approval

The study protocol was approved by the Institutional Review Board of Severance Hospital (4–2022-0645). All study procedures were performed in accordance with relevant guidelines and regulations, including the Declaration of Helsinki. Written informed consent was obtained from all participants prior to enrollment.

### Dietary intake assessment

Dietary intake was assessed using a semi-quantitative food frequency questionnaire (FFQ) developed based on the validated instrument used in the Korean Genome and Epidemiology Study [19, 20]. A relative validity study of this obesity-specific 129-item FFQ against 3-day dietary records demonstrated acceptable validity for ranking individuals according to habitual nutrient intake and good reproducibility in Korean adults with obesity [21]. The FFQ was updated by adding selected food items and incorporating portion-size photographs to facilitate participants’ understanding and improve the accuracy of dietary intake estimation. Participants were asked to recall their average frequency and portion size of food intake over the previous year for each item. To minimize potential bias, researchers reviewed participants’ responses and conducted additional telephone interviews when clarification was needed. Sodium intake was estimated by multiplying the reported frequency and portion size of each food item by its corresponding sodium content derived from Korean food composition tables. The FFQ included major sodium-contributing food items in the Korean diet, such as soups and stews, kimchi, seasoned side dishes, and fermented soybean-based condiments (e.g., doenjang, ganjang, gochujang), thereby capturing the primary sources of dietary sodium in this population. Discretionary salt use, such as salt added during cooking or the table, was not separately captured. Accordingly, FFQ-estimated sodium intake should be interpreted as reflecting the relative ranking of habitual sodium intake rather than precise estimates of absolute sodium intake. FFQ-estimated sodium intake was expressed per 1 000 kcal of total energy intake to account for individual differences in energy consumption.

### Physical activity assessment

Physical activity levels were assessed using the short-form version of the International Physical Activity Questionnaire (IPAQ) [22]. Total activity levels were calculated as weekly metabolic equivalent of task (MET)-min and classified according to the WHO criteria as inactive (< 600 MET-min/week), moderately active (600–2 999 MET-min/week), and highly active (≥ 3 000 MET-min/week).

### Outcome definitions

Metabolic diseases were defined as follows: obesity (BMI ≥ 25 kg/m^2^); hypertension (systolic blood pressure (SBP) ≥ 140 mmHg, diastolic blood pressure (DBP) ≥ 90 mmHg, prior physician diagnosis, or use of antihypertensive medication); diabetes mellitus (fasting blood glucose (FBG) ≥ 126 mg/dL, postprandial glucose (PPG) ≥ 200 mg/dL, glycated hemoglobin (HbA1c) ≥ 6.5%, prior physician diagnosis, or use of glucose-lowering medication); dyslipidemia (total cholesterol (TC) ≥ 240 mg/dL, triglycerides (TGs) ≥ 200 mg/dL, high-density lipoprotein cholesterol (HDL-C) < 40 mg/dL, low-density lipoprotein cholesterol (LDL-C) ≥ 160 mg/dL, prior physician diagnosis, or use of lipid-lowering medication); and cardiovascular disease, defined as a history of stroke, transient ischemic attack, myocardial infarction, angina, or heart failure. Disease status was ascertained through a combination of medical record review and in-person or telephone interviews, with diagnoses verified by two independent investigators [17].

### Clinical, biochemical, anthropometric, and image-based measurements

Anthropometric measurements including height, weight, waist circumference (WC), and hip circumference (HC) were obtained using standardized procedures. BMI was calculated as weight divided by height squared (kg/m^2^). Vital signs, including SBP, DBP, and pulse rate, were primarily measured using the right arm after participants were seated at rest. Fasting blood samples were collected to measure FBG, fasting blood insulin (FBI), HbA1c, TC, TGs, HDL-C, and LDL-C. Homeostatic model assessment for insulin resistance (HOMA-IR) was calculated as:

[fasting insulin (μU/mL) × fasting glucose (mg/dL)/405].

Additionally, atherogenic indices were calculated as follows:

- Atherogenic index of plasma (AIP) = log_10_[TGs (mg/dl)/HDL-C (mg/dl)]
- Non-HDL-C = TC—HDL-C (mg/dl)
- Castelli Risk Index-I (CRI-I) = TC (mg/dl)/HDL-C (mg/dl)
- Castelli Risk Index-II (CRI-II) = LDL-C (mg/dl)/HDL-C (mg/dl)

All laboratory analyses were conducted in a central certified laboratory using standardized protocols. Liver fat was assessed using transient elastography (Fibroscan), with hepatic steatosis quantified via the controlled attenuation parameter (CAP). Abdominal fat area was measured by computed tomography (CT) at the L4–L5 vertebral level. All measurements were conducted by trained technicians using standardized protocols.

### Covariates

Covariates were selected a priori based on established risk factors for metabolic diseases and potential confounders of the sodium-metabolic health association, and included age, sex, BMI, smoking status, alcohol consumption, physical activity, and energy intake. Smoking status (yes/no) and alcohol consumption status (yes/no) were assessed via questionnaire, with pack-year and weekly alcohol intake further calculated based on detailed responses, as described in the cohort protocol [17]. For analyses of clinical and metabolic biomarkers, use of metabolic disease-related medications (antihypertensive, lipid-lowering, antidiabetic, and antiobesity agents) was additionally included as a covariate. Medication use was not included as a covariate in logistic regression models for disease prevalence, as it is considered a clinical proxy for the respective disease status rather than an independent confounding factor. BMI was not included as a covariate in models in which obesity or body composition indicators were used as outcomes.

### Statistical analysis

Participants were categorized into sodium intake tertiles based on energy-adjusted values. Differences in baseline characteristics and nutrient intakes across tertiles were examined using analysis of variance (ANOVA) or the chi-squared test, as appropriate. Multivariable logistic regression models were used to estimate odds ratios (ORs) and 95% confidence intervals (CIs) for the prevalence of metabolic diseases across sodium intake tertiles with adjustment for relevant covariates. The lowest tertile of sodium intake was used as the reference category. Trends across tertiles were tested by modeling tertile as an ordinal variable. Two models were used in the logistic regression analyses. Model 1 was unadjusted, and Model 2 was adjusted for age, sex, BMI, smoking status, alcohol consumption, physical activity, and energy intake. Medication use was not included as a covariate in these models, as it is considered a clinical proxy for the respective disease status rather than an independent confounding factor. In addition, differences in clinical and metabolic biomarkers across sodium intake tertiles were examined by sex using ANOVA and analysis of covariance (ANCOVA), with the latter accounting for covariates and use of metabolic disease-related medications. Sex was not included as a covariate in these models, as analyses were conducted separately for men and women. For metabolic disease prevalence analyses, sex was included as a covariate given the uniform diagnostic criteria across sexes. To formally evaluate sex differences in the associations between sodium intake and metabolic biomarkers, sodium × sex interaction terms were tested in multivariable models. To account for the multiple interaction tests across biomarkers, Benjamini–Hochberg FDR-adjusted *p* values were additionally calculated.

## Results

### Participant characteristics across sodium intake tertiles

In total, 633 participants were enrolled between October 2022 and January 2024. After excluding non-obese control participants (*n* = 127) and individuals with implausible energy intake (< 500 or ≥ 5 000 kcal/day) (*n* = 19), 487 participants with obesity were included in the present analysis. Data on disease prevalence were missing for dyslipidemia (*n* = 3), hypertension (*n* = 2), CVD (*n* = 4) (Fig. 1).

The baseline characteristics of the participants by sodium intake tertile are presented in Table 1. The mean participant age was 51.85 ± 11.34 years, and approximately 60% of the participants were male. The BMI was 30.34 ± 4.25 kg/m^2^, confirming that all participants met the obesity criterion. No statistically significant differences were observed across sodium intake tertiles in age, sex, anthropometric measures, or lifestyle factors (all *p* > 0.05).

**Table 1:** Baseline characteristics across sodium intake tertiles

	Total (*n* = 487)	T1 (*n* = 162)	T2 (*n* = 163)	T3 (*n* = 162)	*p*^1)^
Age (years)	51.85 ± 11.34	51.46 ± 12.23	50.83 ± 11.09	53.28 ± 10.57	0.128
Sex^2)^					
Male (%)	293 (60)	93 (57)	103 (63)	97 (60)	0.565
Female (%)	194 (40)	69 (43)	60 (37)	65 (40)	
Height (cm)	167.45 ± 9.04	166.8 ± 9.24	168.13 ± 8.70	167.44 ± 9.19	0.416
Weight (kg)	85.40 ± 15.64	85.38 ± 15.16	86.45 ± 16.37	84.37 ± 15.38	0.490
BMI (kg/m^2^)	30.34 ± 4.25	30.66 ± 4.79	30.43 ± 4.33	29.92 ± 3.52	0.274
Smoking^2)^					
Yes (%)	219 (45)	67 (41)	77 (47)	75 (46)	0.520
No (%)	268 (55)	95 (59)	86 (53)	87 (54)	
Pack-years	17.33 ± 15.00	16.17 ± 15.00	16.61 ± 13.53	19.2 ± 16.48	0.432
Alcohol consumption^2)^					
Yes (%)	289 (59)	93 (57)	100 (61)	96 (59)	0.770
No (%)	198 (41)	69 (43)	63 (39)	66 (41)	
Alcohol intake per week	98.72 ± 140.19	109.32 ± 149.98	87.72 ± 133.41	99.91 ± 137.86	0.563
Antihypertensive medication use^2)^					
Yes (%)	350 (72)	115 (71)	116 (71)	119 (73)	0.859
No (%)	137 (28)	47 (29)	47 (29)	43 (27)	
Lipid-lowering medication use^2)^					
Yes (%)	338 (69)	104 (64)	116 (71)	118 (73)	0.201
No (%)	149 (31)	58 (36)	47 (29)	44 (27)	
Antidiabetic medication use^2)^					
Yes (%)	174 (36)	56 (35)	62 (38)	56 (35)	0.753
No (%)	313 (64)	106 (65)	101 (62)	106 (65)	
Antiobesity medication use^2)^					
Yes (%)	50 (10)	17 (10)	16 (10)	17 (10)	0.973
No (%)	437 (90)	145 (90)	147 (90)	145 (90)	
Physical activity (MET-based, WHO cutoffs)					
Inactive (%)	140 (29)	44 (27)	47 (29)	49 (30)	0.376
Moderate (%)	261 (54)	87 (54)	96 (59)	78 (48)	
Highly active (%)	71 (15)	26 (16)	16 (10)	29 (18)	
No response (%)	15 (3)	5 (3)	4 (2)	6 (4)	

Values are presented as mean ± S.D

^1)^Statistical analysis was performed using one-way ANOVA, ^2)^Statistical analysis was performed using chi-square test

Among all participants, approximately 45% had a history of smoking, and 59% reported alcohol consumption. Concerning medication use, 72% were taking antihypertensive medications, 69% were on lipid-lowering medications, 36% were using antidiabetic agents, and 10% were prescribed antiobesity medications. According to MET-based physical activity assessment, 54% of participants engaged in moderate-intensity physical activity, 29% were classified as inactive, and 15% as highly active.

Table 2 summarizes dietary nutrient intake across sodium intake tertiles. The mean sodium intake was 3 046.52 ± 1 388.40 mg in T1, 4 370.48 ± 1 769.62 mg in T2, and 5 574.79 ± 2 433.76 mg in T3. Tertile cut-off values for energy-adjusted sodium intake were as follows: T1 < 1 798.43 mg/1 000 kcal; T2 1 798.43–2 174.58 mg/1 000 kcal; T3 ≥ 2 174.59 mg/1 000 kcal. Intakes of carbohydrate, fiber, lipid, saturated fat, protein, vitamin A, and vitamin E differed significantly across tertiles (all *p* < 0.05), with T3 generally showing higher intakes than T1. Carbohydrate intake was highest in T2. There were no significant differences in total energy, sugar, or flavonoid intakes across tertile groups.

**Table 2:** Dietary nutrient intake across sodium intake tertiles

	Total (*n* = 487)	T1 (*n* = 162)	T2 (*n* = 163)	T3 (*n* = 162)	*p*^1)^
Calorie (kcal)	2 131.70 ± 886.52	1 996.51 ± 844.70	2 213.82 ± 885.85	2 184.25 ± 917.21	0.057
Carbohydrate (g)	289.09 ± 117.43	289.54 ± 121.70^a^	300.36 ± 118.48^b^	277.31 ± 111.47^c^	<.0001^***^
Fiber (g)	26.10 ± 12.00	22.50 ± 10.94^a^	26.19 ± 11.85^a^	29.61 ± 12.19^b^	<.0001^***^
Sugar (g)	51.54 ± 30.52	48.86 ± 33.95	55.30 ± 33.15	50.43 ± 23.05	0.462
Lipid (g)	63.08 ± 33.80	52.47 ± 29.52^a^	65.64 ± 31.80^b^	71.11 ± 37.09^c^	<.0001^***^
Saturated fat (g)	17.33 ± 9.92	15.01 ± 9.38^a^	18.14 ± 9.57^b^	18.83 ± 10.43^b^	<.0001^***^
Protein (g)	85.05 ± 40.29	70.93 ± 32.06^a^	87.85 ± 38.53^b^	96.34 ± 45.19^c^	<.0001^***^
Sodium (mg)	4 330.68 ± 2 170.44	3 046.52 ± 1 388.40^a^	4 370.48 ± 1 769.62^b^	5 574.79 ± 2 433.76^c^	<.0001^***^
Vitamin A (μg RAE)	458.51 ± 245.23	373.77 ± 225.54^a^	464.19 ± 241.99^b^	537.53 ± 241.26^c^	<.0001^***^
Vitamin E (mg α-TE)	7.40 ± 3.50	6.11 ± 3.05^a^	7.56 ± 3.24^b^	8.53 ± 3.75^c^	<.0001^***^
Flavonoid (mg)	320.29 ± 203.05	332.95 ± 225.23	317.25 ± 208.85	310.70 ± 171.96	0.039^*^

Values are presented as mean ± S.D

Tertile cut-off values for energy-adjusted sodium intake were as follows: T1 < 1 798.43 mg/1 000 kcal; T2 1 798.43–2 174.58 mg/1 000 kcal; T3 ≥ 2 174.59 mg/1 000 kcal

^*^ *p* < 0.05, *p* < 0.001. Different superscript indicate significant differences between groups in the post hoc test (*p* < 0.05)

^1)^Statistical analysis was performed using one-way ANOVA

### Metabolic disease prevalence across sodium intake

Table 3 summarizes the prevalence and ORs of chronic metabolic diseases across sodium intake tertiles. No statistically significant associations were observed between sodium intake and the prevalence of T2DM, dyslipidemia, hypertension, or CVD in either unadjusted or adjusted models (all *p* > 0.05). The distribution of CVD components (stroke, transient ischemic attack, myocardial infarction, angina, and heart failure) across sodium intake tertiles is presented in Supplementary Table 1.

**Table 3:** Odds ratios for chronic metabolic disease prevalence across sodium intake tertiles

	T1	T2	T3	*p*
T2DM				
Cases/Total (%)	76/162 (47)	82/163 (50)	86/162 (53)	
Model 1, OR (95% CI)	Ref	1.15 (0.74, 1.77)	1.28 (0.83–1.98)	0.540
Model 2, OR (95% CI)	Ref	1.13 (0.72, 1.76)	1.30 (0.83–2.03)	0.519
Dyslipidemia				
Cases, n (%)	122/161 (76)	132/162 (81)	133/161 (83)	
Model 1, OR (95% CI)	Ref	1.41 (0.82, 2.40)	1.52 (0.88, 2.62)	0.262
Model 2, OR (95% CI)	Ref	1.42 (0.80, 2.53)	1.36 (0.76, 2.45)	0.421
Hypertension				
Cases, n (%)	115/161 (71)	115/162 (71)	120/162 (74)	
Model 1, OR (95% CI)	Ref	0.98 (0.61, 1.58)	1.14 (0.70, 1.87)	0.799
Model 2, OR (95% CI)	Ref	0.87 (0.51, 1.47)	0.98 (0.57, 1.67)	0.854
CVD				
Cases, n (%)	23/161 (14)	22/163 (13)	24/159 (15)	
Model 1, OR (95% CI)	Ref	0.94 (0.50, 1.76)	1.07 (0.57, 1.98)	0.920
Model 2, OR (95% CI)	Ref	1.00 (0.50, 1.99)	1.02 (0.52, 2.00)	0.998

*CI* confidence interval, *T2DM* type 2 diabetes mellitus, *CVD* cardiovascular disease

Statistical analysis was performed using multivariable logistic regression

Model 1: Crude logistic regression model

Model 2: Adjusted for age, sex, BMI, energy intake, smoking history, alcohol consumption, and physical activity level (categorized by MET cutoffs)

### Clinical, biochemical, anthropometric, and image-based parameters

Clinical, biochemical, anthropometric, and image-based parameters according to sodium intake tertiles, stratified by sex, are presented in Table 4. Covariate-adjusted means and 95% CI for these parameters are provided in Supplementary Table 2. Among male participants, no statistically significant associations were observed between sodium intake and any clinical or metabolic biomarker in either unadjusted or adjusted models (all *p* > 0.05).

**Table 4:** Clinical and metabolic biomarkers according to sodium intake tertiles, stratified by sex

	Male					Female				
	T1 (*n* = 93)	T2 (*n* = 103)	T3 (*n* = 97)	*p*^1)^	*p*^2)^	T1 (*n* = 69)	T2 (*n* = 60)	T3 (*n* = 65)	*p*^1)^	*p*^2)^
Body weight (kg)^†^	89.96 ± 14.30	91.94 ± 16.31	89.80 ± 15.18	0.547	0.941	79.20 ± 14.14	77.02 ± 11.54	76.27 ± 11.73	0.376	0.230
BMI (kg/m^2^)^†^	30.12 ± 3.73	30.65 ± 4.58	29.96 ± 3.63	0.450	0.686	31.39 ± 5.87	30.06 ± 3.87	29.85 ± 3.36	0.107	0.030^*^
WC (cm)^†^	99.15 ± 10.88	99.49 ± 12.45	99.60 ± 9.80	0.959	0.670	97.28 ± 11.19	96.58 ± 8.91	95.65 ± 9.47	0.639	0.389
HC (cm)^†^	104.76 ± 7.56	105.62 ± 8.41	104.82 ± 7.38	0.691	0.961	104.69 ± 8.76	103.37 ± 6.30	102.57 ± 6.30	0.241	0.157
SBP (mmHg)	133.87 ± 14.18	133.19 ± 14.64	130.68 ± 13.31	0.256	0.258	129.03 ± 15.25	130.57 ± 14.44	129.54 ± 14.2	0.835	0.709
DBP (mmHg)	82.43 ± 10.18	82.57 ± 9.90	81.69 ± 10.50	0.809	0.876	77.75 ± 11.12	79.10 ± 9.17	76.68 ± 8.99	0.391	0.474
FBG (mg/dL)	112.26 ± 25.69	114.12 ± 26.20	112.29 ± 24.89	0.843	0.774	106.97 ± 24.88	114.08 ± 24.33	106.02 ± 21.08	0.116	0.376
FBI (μU/mL)	15.19 ± 9.57	15.89 ± 10.90	14.61 ± 14.78	0.756	0.776	17.49 ± 11.15	14.19 ± 9.09	14.17 ± 10.21	0.110	0.497
HOMA-IR	4.25 ± 2.95	4.57 ± 3.65	4.22 ± 5.00	0.791	0.865	4.70 ± 3.18	4.11 ± 3.25	3.91 ± 3.67	0.388	0.809
HbA1c (%)	6.25 ± 0.83	6.23 ± 0.75	6.30 ± 1.00	0.853	0.777	6.30 ± 0.85	6.33 ± 0.96	6.27 ± 1.05	0.944	0.705
TC (mg/dL)	160.27 ± 36.74	160.22 ± 41.37	152.56 ± 33.99	0.262	0.859	161.93 ± 37.12	173.73 ± 40.90	168.82 ± 46.06	0.268	0.085
TGs (mg/dL)	175.35 ± 113.21	155.82 ± 83.17	149.05 ± 77.35	0.129	0.163	137.30 ± 62.97	149.62 ± 70.62	130.31 ± 69.49	0.276	0.262
HDL-C (mg/dL)	45.43 ± 8.45	47.16 ± 11.44	46.74 ± 9.42	0.458	0.563	52.62 ± 12.07	51.93 ± 12.20	53.78 ± 12.76	0.697	0.821
LDL-C (mg/dL)	91.29 ± 34.93	93.44 ± 38.01	85.60 ± 32.09	0.273	0.714	90.94 ± 31.56^a^	101.80 ± 36.66^b^	96.92 ± 40.03^ab^	0.234	0.038^*^
AIP	0.53 ± 0.27	0.48 ± 0.24	0.47 ± 0.24	0.226	0.303	0.39 ± 0.25	0.43 ± 0.23	0.35 ± 0.25	0.175	0.199
Non-HDL-C	114.95 ± 36.85	113.07 ± 41.30	105.82 ± 33.42	0.208	0.837	109.30 ± 33.77^a^	121.80 ± 38.15^b^	115.03 ± 44.36^ab^	0.194	0.030^*^
CRI-I	3.63 ± 1.05	3.54 ± 1.12	3.36 ± 0.92	0.199	0.721	3.18 ± 0.87^a^	3.46 ± 0.94^b^	3.26 ± 1.03^ab^	0.220	0.044^*^
CRI-II	2.06 ± 0.86	2.09 ± 0.97	1.89 ± 0.79	0.237	0.790	1.79 ± 0.68^a^	2.05 ± 0.81^b^	1.89^ab^ ± 0.87^ab^	0.179	0.018^*^

*Abbreviations*: *BMI* body mass index, *WC* waist circumference, *HC* hip circumference, *SBP* systolic blood pressure, *DBP* diastolic blood pressure, *FBG* fasting blood glucose, *FBI* fasting blood insulin, *HOMA-IR* homeostatic model assessment of insulin resistance, *TC* total cholesterol, *TG* triglyceride, *HDL-C* high density lipoprotein cholesterol, *LDL-C* low density lipoprotein cholesterol, *AIP* atherogenic index of plasma = log_10_(TG/HDL-C), *Non-HDL-C* TC-HDL-C, *CRI-I* TC/HDL-C, CRI-II = LDL-C/HDL-C

^1)^One-way ANOVA

^2)^ANCOVA adjusted for age, BMI, energy intake, smoking status, alcohol consumption, physical activity level (categorized by MET cutoffs), and use of metabolic disease-related medications followed by Tukey’s post hoc test

^†^BMI was not included as a covariate in models when the outcome was directly related to adiposity

Data were available for the following number of participants: WC and HC, *n* = 483; SBP and DBP, *n* = 486; FBG, *n* = 485; FBI, *n* = 476; HOMA-IR, *n* = 475; HbA1c, *n* = 478; TC and TG, *n* = 486; HDL-C, LDL-C, AIP, Non-HDL, CRI-I, and CRI-II, *n* = 485. All analyses used a complete-case approach

^*^ *p* < 0.05. Different superscript indicate significant differences between groups in the post hoc test (*p* < 0.05)

Among female participants, adjusted analyses revealed statistically significant associations between sodium intake and several lipid-related markers. LDL-C, non-HDL-C, CRI-I, and CRI-II all showed significant differences across sodium intake tertiles (*p* = 0.038, *p* = 0.030, *p* = 0.044, and *p* = 0.018, respectively), with T2 showing the highest values (T2 > T3 > T1 in post-hoc comparisons). TC showed a marginal trend in the adjusted model (*p* = 0.085). BMI also differed significantly across sodium intake tertiles (*p* = 0.030); however, no pairwise differences remained significant after post-hoc adjustment.

Formal sex-by-sodium interaction testing was conducted for all biomarkers; however, no statistically significant interactions were observed (Supplementary Table 3).

Other clinical, anthropometric, and image-based parameters showed no statistically significant differences across sodium intake tertiles.

## Discussion

In this cohort of Korean adults with obesity and chronic metabolic conditions, participants consumed approximately 1.5–2.8 times more sodium than the WHO recommended intake. Despite this substantially elevated sodium consumption no statistically significant associations were observed between sodium intake and the prevalence of T2DM, dyslipidemia, hypertension, or CVD. However, sex-stratified biomarker analyses revealed a consistent pattern in women, with LDL-C, non-HDL-C, CRI-I, and CRI-II showing higher values compared to T1, with the most pronounced elevation observed in T2. Of note, the lipid-related markers showing consistent patterns in women—including LDL-C, non-HDL-C, CRI-I, and CRI-II—are recognized as integrated indicators of cardiometabolic risk that capture atherogenic lipoprotein burden beyond conventional LDL-C measurement. Non-HDL-C reflects the TC carried by all atherogenic lipoproteins [23], while CRI-I and CRI-II provide estimates of the balance between atherogenic and antiatherogenic lipoproteins [24]. These indices have been associated with increased cardiovascular risk in observational studies, particularly in individuals with metabolic comorbidities. The observation that these cardiometabolic markers showed directional patterns across sodium tertiles in women, even in the absence of significant disease-level associations, raises the possibility that sodium-associated metabolic disturbances may be more apparent at the biochemical level prior to the development of overt cardiometabolic disease. Although formal sex-by-sodium interaction testing did not confirm statistically significant interactions, the directional consistency of these lipid-related findings across multiple markers suggests a possible sex-specific susceptibility to sodium-associated adverse lipid profiles that warrants further investigation.

The biological plausibility of sodium-associated lipid disturbances has been suggested by several preclinical mechanisms. High salt intake has been closely linked to adipose tissue dysfunction and inflammation [8]. Excessive salt exposure promotes adipogenic and lipogenic gene expression (PPAR-γ, C/EBPα, SREBP1) and suppresses lipolytic pathways such as AMPK, potentially resulting in increased lipid storage and inflammation within adipose tissue [8]. In animal models, dietary salt restriction has been shown to attenuate visceral adipose tissue inflammation and cardiac oxidative stress [25]. Consistent with these mechanistic observations, clinical evidence also supports a possible relationship between sodium intake and lipid metabolism. In a weight reduction trial, serum lipid levels returned to normal in most obese patients following sodium-restriction [26]. Likewise, in a cross-sectional study of hypertensive women, variations in blood lipid levels were significantly explained by age and sodium intake among those with excess body weight [27].

Despite this mechanistic plausibility, no statistically significant associations were observed between sodium intake and the prevalence of major metabolic diseases in the present study. This may partly reflect the nature of the study population, in which nearly all participants had pre-existing metabolic conditions and were receiving pharmacological treatment, potentially reducing the variability in disease prevalence across sodium intake levels. Disease prevalence represents an end-stage manifestation of cumulative metabolic dysfunction, and detecting sodium-associated differences at this stage may be inherently limited in a clinically treated high-risk cohort. These findings suggest that cross-sectional associations between sodium intake and metabolic outcomes may be more apparent at the biochemical level, particularly in lipid-related markers, before clinically manifest disease develops. Whether dietary sodium management may benefit high-risk individuals prior to the onset of overt metabolic disease warrants confirmation in future longitudinal and intervention studies.

The sex-stratified lipid findings in the present study may reflect a greater responsiveness among women with obesity to sodium-associated metabolic disturbances. This may be partly attributable to sex-specific differences in salt sensitivity, hormonal regulation, and adipose tissue physiology. In general, women tend to be more salt-sensitive than men, and their risk of CVD begins to increase at lower SBP levels [28]. Hormonal changes associated with menopause, particularly the decline in estrogen levels, may promote central adiposity, insulin resistance, altered blood pressure regulation, and adverse lipid profiles, thereby increasing susceptibility to sodium-associated metabolic disturbances [28, 29]. Consistent with this notion, clinical evidence suggests that estrogen replacement therapy can attenuate salt sensitivity and improve blood pressure regulation in response to salt intake among postmenopausal women [30]. Given that the mean age of the study population was 51.85 ± 11.34 years, hormonal transitions associated with aging may have influenced the observed sex-specific patterns.

This study represents the first analysis from the NAINOC cohort, a prospective cohort designed to comprehensively examine the clinical, nutritional, and metabolic characteristics of Korean adults with obesity and related chronic metabolic conditions. As such, it provides baseline characterization of habitual sodium intake and its cross-sectional associations with metabolic outcomes in this high-risk population. The calculation of atherogenic indices, including AIP, non-HDL-C, CRI-I, and CRI-II, allowed for a more comprehensive assessment of CVD risk beyond conventional lipid markers. Furthermore, sex-specific analyses provided exploratory insights into possible sex-specific patterns that may inform the design of future longitudinal analyses within this cohort.

However, several limitations should be acknowledged. First, because of its cross-sectional design based on baseline data, causal inferences regarding sodium intake and metabolic outcomes cannot be drawn. As this study is part of an ongoing prospective cohort, future longitudinal analyses using follow-up data will help clarify these relationships more robustly. Second, sodium intake was estimated using an FFQ, which may be subject to recall bias and may not accurately capture discretionary salt use or daily variation. A dedicated validation study of this obesity-specific 129-item FFQ has demonstrated acceptable relative validity for ranking individuals and good reproducibility [21]; however, the energy-adjusted correlation for sodium was somewhat lower than for other nutrients, and FFQ-estimated sodium intake should be interpreted as reflecting the relative ranking of habitual sodium intake rather than precise estimates of absolute intake. Third, residual confounding from variables not included in the adjustment models cannot be entirely excluded. Socioeconomic status data were not collected as part of the cohort protocol, and potassium intake and overall diet quality were unavailable in the food composition database linked to the FFQ, precluding their inclusion as covariates. Furthermore, sodium density may partly reflect broader dietary patterns. Accordingly, the observed associations should be interpreted with caution, as residual confounding by overall diet quality or major food sources cannot be ruled out. Fourth, this study examined multiple metabolic outcomes and biomarkers simultaneously, which increases the possibility of chance findings. As no formal multiple testing correction was applied to the main analyses, results should be considered exploratory and interpreted in the context of the overall pattern of associations rather than relying on isolated significant findings. Fifth, the cohort was enriched with individuals at higher metabolic risk, which may limit generalizability but enhances the relevance of the findings to high-risk groups. Furthermore, as enrollment was conditional on having at least one metabolic disease, the possibility of collider bias cannot be excluded. Given these pre-existing metabolic conditions, participants may have already modified their sodium intake based on medical advice, and reverse causation therefore cannot be entirely ruled out.

Despite several limitations, this study provides exploratory evidence on the cross-sectional associations between habitual sodium intake and metabolic outcomes in a clinically characterized Korean cohort with obesity. As the first report from the NAINOC cohort, these findings offer baseline insights into the possible role of sodium intake in cardiometabolic health among high-risk individuals, and may inform the design of future longitudinal analyses within this cohort.

## Supplementary Information

Supplementary Material 1.

## Data Availability

The datasets generated and/or analysed during the current study are not publicly available due to privacy restrictions on individual participant data, but are available from the corresponding author on reasonable request and subject to review and approval by the institutional ethics board.

## Abbreviations

T2DM: Type 2 diabetes mellitus
IR: Insulin resistance
FFQ: Food frequency questionnaire
MET: Metabolic equivalent of task
SBP: Systolic blood pressure
FBG: Fasting blood glucose
TC: Total cholesterol
HDL-C: High-density lipoprotein cholesterol
WC: Waist circumference
FBI: Fasting blood insulin
HOMA-IR: Homeostatic model assessment for insulin resistance
CRI-I: Castelli risk index-I
CT: Computed tomography
ORs: Odds ratios
ANCOVA: Analysis of covariance
CVD: Cardiovascular disease
BMI: Body mass index
IPAQ: International Physical Activity Questionnaire
WHO: World Health Organization
DBP: Diastolic blood pressure
PBG: Postprandial blood glucose
TGs: Triglycerides
LDL-C: Low-density lipoprotein cholesterol
HC: Hip circumference
HbA1c: Glycated hemoglobin
AIP: Atherogenic index of plasma
CRI-II: Castelli risk index-II
ANOVA: Analysis of variance
CIs: Confidence intervals
