# Supplementary material for: Habitual sodium intake and metabolic abnormalities in Korean adults with obesity: a cross-sectional analysis of a prospective cohort
Source: BMC Public Health. 2026 Aug 6;26:3181. doi: 10.1186/s12889-026-28903-y (PMC13729306; doi:10.1186/s12889-026-28903-y)
Supplement: Supplementary file 1 [file 12889_2026_28903_MOESM1_ESM.docx]

**Supplementary Table 1**. Number of participants with each cardiovascular disease component

| CVD component | T1 (n = 161) | T2 (n = 163) | T3 (n = 159) | p-value^1)^ |
| --- | --- | --- | --- | --- |
| Stroke | 4 (2.5) | 1 (0.6) | 1 (0.6) | 0.336 |
| TIA | 0 (0.0) | 0 (0.0) | 1 (0.6) | 0.329 |
| MI | 0 (0.0) | 2 (1.2) | 7 (4.4) | 0.005^2)^ |
| Angina | 15 (9.3) | 13 (8.0) | 13 (8.2) | 0.910^2)^ |
| Heart failure | 8 (5.0) | 8 (4.9) | 6 (3.8) | 0.846 |
| Total CVD^†^ | 24 (14.9) | 22 (13.5) | 24 (15.1) | 0.920 |

^1)^Chi-square test, ^2)^Fisher’s exact test

^†^Total CVD represents the number of participants with at least one cardiovascular disease component. Participants with multiple components are counted once.

**Supplementary Table 2**. Adjusted means and 95% confidence intervals for clinical and metabolic biomarkers according to sodium intake tertiles, stratified by sex.

|  | Male | | | | Female | | | |
| --- | --- | --- | --- | --- | --- | --- | --- | --- |
|  | T1 (n = 93) | T2 (n = 103) | T3 (n = 97) | *p*^1)^ | T1 (n = 69) | T2 (n = 60) | T3 (n = 65) | *p*^1)^ |
| Body weight (kg)^†^ | 90.67 (88.01, 93.32) | 90.88 (88.38, 93.39) | 90.25 (87.67, 92.83) | 0.941 | 79.5 (76.73, 82.27) | 76.38 (73.43, 79.32) | 76.54 (73.67, 79.41) | 0.230 |
| BMI (kg/m^2^)^†^ | 30.3 (29.56, 31.03) | 30.44 (29.75, 31.13) | 30.01 (29.29, 30.72) | 0.686 | 31.61 (30.56, 32.65) | 29.84 (28.73, 30.95) | 29.82 (28.74, 30.90) | 0.030^*^ |
| WC (cm)^†^ | 99.84 (97.84, 101.84) | 98.72 (96.82, 100.62) | 99.76 (97.8, 101.72) | 0.670 | 97.76 (95.46, 100.07) | 96.06 (93.66, 98.47) | 95.54 (93.20, 97.89) | 0.389 |
| HC (cm)^†^ | 105.17 (103.80, 106.54) | 105.15 (103.85, 106.46) | 104.92 (103.58, 106.27) | 0.961 | 104.85 (103.17, 106.53) | 103.04 (101.29, 104.80) | 102.61 (100.90, 104.32) | 0.157 |
| SBP (mmHg) | 134.07 (131.21, 136.94) | 132.99 (130.29, 135.68) | 130.77 (127.99, 133.55) | 0.258 | 128.53 (124.96, 132.10) | 130.69 (126.93, 134.45) | 129.95 (126.28, 133.62) | 0.709 |
| DBP (mmHg) | 82.67 (80.60, 84.74) | 82.14 (80.19, 84.09) | 81.93 (79.92, 83.94) | 0.876 | 77.70 (75.25, 80.15) | 79.03 (76.45, 81.61) | 76.80 (74.28, 79.32) | 0.474 |
| FBG (mg/dL) | 112.85 (107.73, 117.98) | 114.17 (109.30, 119.04) | 111.60 (106.57, 116.64) | 0.774 | 107.41 (102.18, 112.64) | 112.12 (106.62, 117.63) | 107.36 (101.99, 112.73) | 0.376 |
| FBI (μU/mL) | 15.53 (13.46, 17.60) | 15.53 (13.54, 17.52) | 14.62 (12.57, 16.67) | 0.776 | 16.43 (14.09, 18.77) | 14.55 (12.16, 16.94) | 14.83 (12.47, 17.19) | 0.497 |
| HOMA-IR | 4.36 (3.65, 5.08) | 4.46 (3.77, 5.15) | 4.19 (3.48, 4.90) | 0.865 | 4.44 (3.65, 5.24) | 4.11 (3.3, 4.92) | 4.12 (3.32, 4.92) | 0.809 |
| HbA1c (%) | 6.29 (6.14, 6.45) | 6.22 (6.07, 6.36) | 6.27 (6.11, 6.42) | 0.777 | 6.34 (6.15, 6.53) | 6.23 (6.03, 6.43) | 6.31 (6.11, 6.5) | 0.705 |
| TC (mg/dL) | 158.17 (151.82, 164.53) | 158.74 (152.74, 164.75) | 156.39 (150.15, 162.63) | 0.859 | 161.64 (152.98, 170.31) | 175.93 (166.80, 185.05) | 167.1 (158.19, 176.00) | 0.085 |
| TGs (mg/dL) | 175.24 (156.18, 194.30) | 153 (135.10, 170.90) | 152.31 (133.72, 170.91) | 0.163 | 135.12 (119.19, 151.06) | 150.28 (133.50, 167.07) | 132.01 (115.64, 148.38) | 0.262 |
| HDL-C (mg/dL) | 45.65 (43.62, 47.68) | 47.18 (45.28, 49.08) | 46.52 (44.55, 48.50) | 0.563 | 52.88 (49.85, 55.91) | 52.02 (48.83, 55.21) | 53.43 (50.32, 56.55) | 0.821 |
| LDL-C (mg/dL) | 88.86 (83.21, 94.51) | 91.96 (86.66, 97.27) | 89.65 (84.13, 95.16) | 0.714 | 90.47 (83.4, 97.54) | 103.88 (96.44, 111.32) | 95.51 (88.25, 102.77) | 0.038^*^ |
| AIP | 0.52 (0.47, 0.58) | 0.47 (0.42, 0.52) | 0.48 (0.43, 0.53) | 0.303 | 0.38 (0.32, 0.43) | 0.43 (0.37, 0.49) | 0.35 (0.3, 0.41) | 0.199 |
| Non-HDL | 112.50 (106.28, 118.73) | 111.59 (105.74, 117.44) | 109.89 (103.81, 115.96) | 0.837 | 108.76 (100.97, 116.55) | 123.91 (115.70, 132.11) | 113.66 (105.66, 121.66) | 0.030^*^ |
| CRI-I | 3.57 (3.39, 3.75) | 3.50 (3.33, 3.67) | 3.46 (3.29, 3.64) | 0.721 | 3.16 (2.96, 3.36) | 3.51 (3.3, 3.72) | 3.23 (3.03, 3.44) | 0.044^*^ |
| CRI-II | 2.00 (1.85, 2.14) | 2.05 (1.92, 2.19) | 1.99 (1.85, 2.13) | 0.790 | 1.77 (1.62, 1.93) | 2.09 (1.93, 2.26) | 1.87 (1.71, 2.03) | 0.018^*^ |

**Abbreviations:** BMI, body mass index; WC, waist circumference; HC, hip circumference; SBP, systolic blood pressure; DBP, diastolic blood pressure; FBG, fasting blood glucose; FBI, fasting blood insulin; HOMA-IR, homeostatic model assessment of insulin resistance; TC, total cholesterol; TG, triglyceride; HDL-C, high density lipoprotein cholesterol; LDL-C, low density lipoprotein cholesterol; AIP, atherogenic index of plasma = log_10_(TG/HDL-C); Non-HDL-C = TC-HDL-C; CRI-I = TC/HDL-C; CRI-II = LDL-C/HDL-C

Adjusted means and 95% confidence intervals were estimated as population marginal means (least-squares means with the observed-margins option) from ANCOVA models, weighting categorical covariates by their observed distribution in the sample. Models were adjusted for age, BMI, energy intake, smoking status, alcohol consumption, physical activity level (categorized by MET cutoffs), and use of metabolic disease-related medications.

^†^BMI was not included as a covariate in models when the outcome was directly related to adiposity.

^*^*p* < 0.05

**Supplementary Table 3**. Sex-by-sodium intake interaction test results for clinical and metabolic biomarkers.

| Biomarker | Interaction estimate (95% CI) | *p*-interaction^1)^ | FDR-adjusted *p*^2)^ |
| --- | --- | --- | --- |
| Body weight^†^ | -0.37 (-3.16, 2.41) | 0.792 | 0.975 |
| BMI^†^ | -0.45 (-1.32, 0.43) | 0.320 | 0.720 |
| WC^†^ | -0.42 (-2.61, 1.77) | 0.706 | 0.975 |
| HC^†^ | -0.54 (-2.07, 0.99) | 0.488 | 0.975 |
| SBP | 2.42 (-0.74, 5.58) | 0.133 | 0.552 |
| DBP | -0.03 (-2.26, 2.19) | 0.975 | 0.975 |
| FBG | 0.14 (-5.10, 5.39) | 0.958 | 0.975 |
| FBI | -0.40 (-2.66, 1.86) | 0.731 | 0.975 |
| HOMA-IR | -0.07 (-0.84, 0.70) | 0.862 | 0.975 |
| HbA1c | -0.01 (-0.18, 0.17) | 0.943 | 0.975 |
| TC | 5.32 (-2.02, 12.66) | 0.155 | 0.552 |
| TGs | 9.35 (-8.93, 27.63) | 0.315 | 0.720 |
| HDL-C | -0.34 (-2.76, 2.09) | 0.786 | 0.975 |
| LDL-C | 4.26 (-2.04, 10.55) | 0.184 | 0.552 |
| AIP | 0.01 (-0.04, 0.07) | 0.663 | 0.975 |
| Non-HDL | 5.65 (-1.27, 12.58) | 0.110 | 0.552 |
| CRI-I | 0.14 (-0.05, 0.34) | 0.137 | 0.552 |
| CRI-II | 0.11 (-0.04, 0.26) | 0.151 | 0.552 |

^1)^*p*-interaction values were derived from multivariable models including sodium intake tertiles, sex, and their interaction term (sodium tertile×sex), adjusted for age, BMI, energy intake, smoking status, alcohol consumption, physical activity level (categorized by MET cutoffs), and use of metabolic disease-related medications.

^2)^FDR-adjusted p values were calculated using the Benjamini-Hochberg method.

^†^BMI was not included as covariate in models when the outcome was directly related to adiposity.
